# Distinct Aging-Vulnerable and -Resilient Trajectories of Specific Motor Circuit Functions in Oxidation- and Temperature-Stressed *Drosophila*

**DOI:** 10.1523/ENEURO.0443-21.2021

**Published:** 2022-01-07

**Authors:** Atulya Iyengar, Hongyu Ruan, Chun-Fang Wu

**Affiliations:** 1 Department of Biology, University of Iowa, Iowa City, Iowa 52242; 2 Iowa Neuroscience Institute, University of Iowa, Iowa City, Iowa 52242; 3 Interdisciplinary Graduate Program in Neuroscience, University of Iowa, Iowa City, Iowa 52242

**Keywords:** escape circuit, flight, habituation, oxidative stress, seizure, temperature stress

## Abstract

In *Drosophila*, molecular pathways affecting longevity have been extensively studied. However, corresponding neurophysiological changes underlying aging-related functional and behavioral deteriorations remain to be fully explored. We examined different motor circuits in *Drosophila* across the life span and uncovered distinctive age-resilient and age-vulnerable trajectories in their established functional properties. In the giant fiber (GF) and downstream circuit elements responsible for the jump-and-flight escape reflex, we observed relatively mild deterioration toward the end of the life span. In contrast, more substantial age-dependent modifications were seen in the plasticity of GF afferent processing, specifically in use dependence and habituation properties. In addition, there were profound changes in different afferent circuits that drive flight motoneuron activities, including flight pattern generation and seizure spike discharges evoked by electroconvulsive stimulation. Importantly, in high-temperature (HT)-reared flies (29°C), the general trends in these age-dependent trajectories were largely maintained, albeit over a compressed time scale, lending support for the common practice of HT rearing for expediting *Drosophila* aging studies. We discovered that shortened life spans in *Cu/Zn superoxide dismutase* (*Sod*) mutant flies were accompanied by altered aging trajectories in motor circuit properties distinct from those in HT-reared flies, highlighting differential effects of oxidative versus temperature stressors. This work helps to identify several age-vulnerable neurophysiological parameters that may serve as quantitative indicators for assessing genetic and environmental influences on aging progression in *Drosophila*.

## Significance Statement

Comparisons of the aging trajectories of performance changes of several motor circuits in *Drosophila* revealed remarkably heterogeneous age progressions. We identified “aging-resilient” and “aging-vulnerable” circuits in both normal control and flies with shortened life spans because of either elevated rearing temperature or oxidative stress. Motor circuit components including flight motor neuron and the giant fiber pathway responsible for the escape reflex showed only mild functional decline, whereas distinct trajectories throughout the life span were seen in the flight pattern generator, interneuron inputs to the giant fiber system, and circuits generating seizure discharge patterns. Notably, high-temperature rearing generally compressed aging trajectories while *Sod* mutation-induced oxidative stress led to distinct patterns of motor defects. Together, these results elucidate potentially salient neurophysiological markers for aging in flies.

## Introduction

Aging nervous systems manifest a progressive functional decline. Invertebrate preparations offer experimentally tractable, simpler nervous systems with individually identifiable neurons to reveal the molecular and cellular bases of age-related neurophysiological changes (Janse et al., 1986; Atwood, 1992; Kenyon, 2001; Yeoman and Faragher, 2001; Pletcher et al., 2002; Toivonen and Partridge, 2009). In *Drosophila,* characteristic declines over the life span have been documented in motor coordination (Leffelaar and Grigliatti, 1983; Gargano et al., 2005) and in higher functions, such as learning and memory (Tamura et al., 2003; Yamazaki et al., 2007). However, fewer studies have directly assessed cellular physiological decline underlying changes in behavioral performance across the life span (Martinez et al., 2007; Banerjee et al., 2021). It is important, therefore, to identify the neural circuits and associated behavioral outputs that are prone to age-related modifications and determine whether specific neuronal elements are differentially vulnerable in this process. Given that environmental and genetic contributions must both be considered in studies of the aging process (Miquel et al., 1976; Curtsinger et al., 1995; Mair et al., 2003), it is equally important to examine how age progression of circuit function is differentially modified by extrinsic environmental and intrinsic genetic factors. Such analyses may also uncover salient physiological parameters suitable for the assessment of aging progression.

This study examines several motor pattern circuits in *Drosophila* whose outputs converge on a small subset of identified, well described, flight motor neurons that innervate the indirect flight muscles [dorsal longitudinal muscle a-f (DLMs), each receiving input from a single motor neuron; Levine, 1973]. Thus, the DLM readout, driven by different motor circuits, enabled us to determine how age-dependent changes in different categories of motor performance are modified by either high-temperature (HT) rearing (29°C, leading to a shortened life span) or oxidative stress in short-lived *Cu/Zn superoxide dismutase* (*Sod*) mutants (defective in a major free radical-scavenging enzyme; Campbell et al., 1986; Phillips et al., 1989). Rearing flies at 29°C is a widely adopted protocol for experimental expedience in *Drosophila* aging and neurodegeneration studies (Min and Benzer, 1997; Kang et al., 2002; Simon et al., 2003; Biteau et al., 2010; Sudmeier et al., 2015). Our study directly investigated the impact of rearing at 29°C on different motor functions over the life span, as well as the cellular physiological links between proposed oxidative stress mechanisms (Harman, 1956; Finkel and Holbrook, 2000; Ziegler et al., 2015) and the associated behavioral alterations previously described for *Sod* mutant flies.

We also present an overall assessment of the trajectory of neural circuit performance associated with different categories of motor behaviors along normal and altered life spans. These include flight pattern generation (Levine, 1973; Harcombe and Wyman, 1977), the giant fiber (GF) pathway-mediated jump and flight escape reflex (Tanouye and Wyman, 1980; Gorczyca and Hall, 1984; Engel and Wu, 1992), habituation of the GF pathway escape response to repetitive stimulation (a form of nonassociative learning; Engel and Wu, 1996; Engel et al., 2000), and a stereotypic seizure repertoire triggered by electroconvulsive stimulation (ECS; Pavlidis and Tanouye, 1995; Kuebler and Tanouye, 2000; Lee and Wu, 2002, 2006; Lee et al., 2019). Our analysis has identified age-resilient and age-sensitive circuit properties that are attributable to changes in distinct circuit components. Furthermore, we found that wild-type (WT) flies reared at 29°C manifested accelerated trajectories of age-dependent alterations resembling those at 25°C at a normalized timescale, while *Sod* mutants displayed distinctively altered pattern of aging progression. Our findings also identify several neurophysiological parameters that could serve as quantitative indices for assessing age-related functional decline. These findings have been reported in part in PhD dissertations (Ruan, 2008; Iyengar, 2016) and in abstract form (Wu and Ruan, 2008; Ruan and Wu, 2009; Iyengar et al., 2010).

## Materials and Methods

### Fly stock maintenance and life-span assays

**Figure 1.**
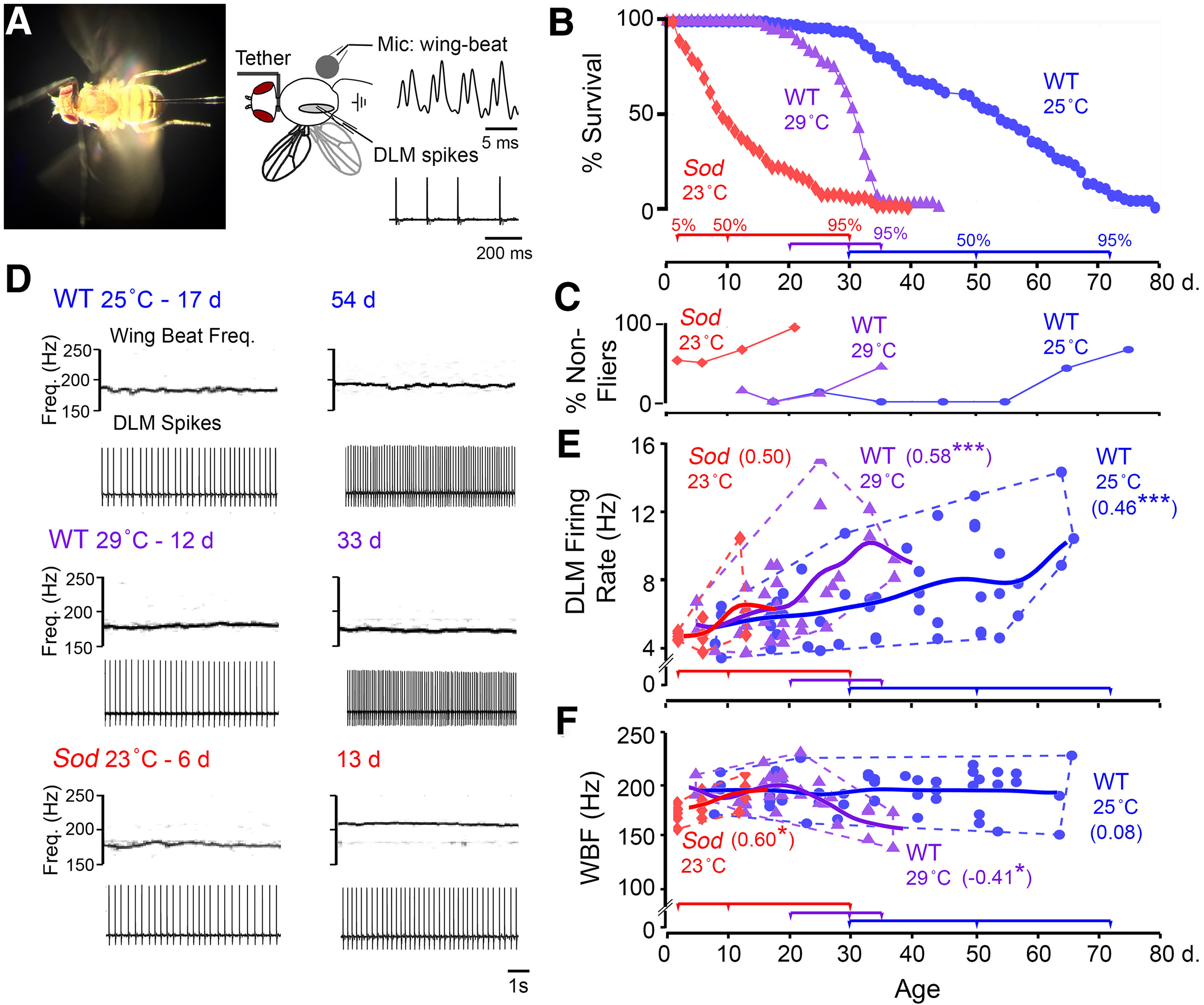
Flight motor performance across the life span of WT control and *Sod* mutant flies. ***A***, Left, Photograph of tethered fly preparation used in the flight and electrophysiological assays. Spiking activity in the DLM was monitored by a tungsten microelectrode, and acoustic signals generated by wing beats were picked up via a microphone placed below the fly. Right, A schematic drawing of the preparation and sample waveforms of the respective signals. ***B***, Life-span curves of WT flies (reared at 25°C, blue circles, *n* = 110; and reared at 29°C, purple triangles, *n* = 104), and *Sod* mutants (red diamonds, reared at 23°C, *n* = 134). Colored timelines below indicate the ages corresponding to 5, 50, and 95% mortality for the respective curves (Extended Data Figure 1-1; log-transformed mortality curves). ***C***, The proportion of nonfliers (for definition, see Materials and Methods) across the life span (*n* > 10 for each point). ***D***, Representative spectrograms of the microphone signal (top panels) indicating the WBF and corresponding traces of DLM spiking (bottom panels) during sustained flight in younger (left column) and aged flies (right column) from the populations tested. ***E***, ***F***, Scatterplots of the DLM firing rate (***E***) and WBF (***F***) during sustained flight in 25°C-reared and 29°C-reared WT flies (blue circles and purple triangles, respectively), and in *Sod* mutants (red diamonds). Trend lines represent a Gaussian kernel running average of the age trajectory for the three populations (kernel window sizes of 10, 6, and 6 d, respectively). The age-dependent *r*_s_ is indicated for each population (*n* = 48, 33, and 15 flies for WT 25°C-reared, WT 29°C-reared, and *Sod*, respectively). For this and following figures: *p < 0.05, **p < 0.01, ***p < 0.001.

The *Canton-S* WT strain and *Sod* mutant allele *Sod^1^* were maintained on standard cornmeal medium (Frankel and Brousseau, 1968). Originally isolated in an ethyl methanesulfonate mutagenesis screen as *l-108* (Campbell et al., 1986), the *Sod^1^* allele (also known as *Sod^n108^*) was recombined on a *red* chromosome background (Phillips et al., 1989) and kept as a balanced stock *Sod^1^ red*/TM3, *Sb Ser.* Among *Sod* alleles previously studied (e.g., *Sod^x39^*, *Sod^n58^*, *Sod^n64^* all on the *red* background; *Sod^21^* on a *white* background), *Sod^1^* was selected for this study because this allele has been well characterized and could be more readily managed to generated sufficient numbers of flies for cross-sectional comparisons across the life span (Ruan and Wu, 2008; Şahin et al., 2017). For collecting rare homozygous offspring of *Sod* flies, the stock was grown in half-pint bottles to encourage reproduction. Newly eclosed adults were collected under CO_2_ anesthesia (WT every 1–2 d, *Sod* flies every 2 d) to assemble sufficient sample sizes to initiate life-span assays. Flies were distributed into food vials (10–14 flies per vial for WT, 7–10 flies for *Sod*) and were transferred to fresh vials every 2 d. All vials were prewarmed to the corresponding temperature before fly transfers. Foam plugs were used, and the vials were kept horizontally to avoid weaker flies being accidentally stuck to food or cotton plugs. Flies were maintained in incubators set at 25°C and 29°C or 23°C, were kept at the ambient humidity range of ∼30–70%, and under 12 h light/dark conditions. Survivors were counted, and dead flies were removed daily. When reported as a function of the percentage of mortality, flies were sampled within 1 d of the age corresponding with percentage of mortality (see Results; Fig. 1*A*).

10.1523/ENEURO.0443-21.2021.f1-1 Figure 1-1 Log-transformed mortality rate. The mortality rate is defined as the negative slope of the survival curve (*S*) [i.e. mortality rate = –1(*S*(*t*_0_) – *S*(*t*_1_))/(*t*_0_ – *t*_1_), for the interval between time points *t*_0_ and *t*_1_]. The log-transformed mortality rates corresponding to the survival curves in Figure 1*B* are plotted. Solid lines correspond to the linear Gompertz fit of the mortality rate distribution [i.e., ln (mortality rate) = ln (*A*) + *Gt*]. The fit parameters *A* and *G* for each distribution are indicated. For CS 25°C-reared and 29°C-reared flies, the Gompertz line is a reasonable fit for the mortality rate over most of the survival curve. However, there is a marked deviation observed in the oldest flies (∼95% survival, shaded region). The *Sod* life-span curve displays a distinctive negative mortality rate slope, implying a potentially distinct set of factors that contribute to mortality in these individuals. Download Figure 1-1, TIF file.

### Flight assessment and indirect flight muscle recording

Preparation, stimulation, recording, and analysis of DLM responses have been described previously (Engel and Wu, 1992; Lee and Wu, 2002; Iyengar and Wu, 2014). Flies were anesthetized briefly on ice or by light ether exposure and glued to a metal wire between the head and thorax using a nitrocellulose or cyanoacrylate-based adhesive (Gorczyca and Hall, 1984). Mounted flies were allowed at least 30 min rest in a humid chamber before recording. All electrophysiological and behavioral experiments were conducted at room temperature (21–23°C).

Flights were triggered via a gentle 500 ms air puff generated by an aquarium air pump (Whisper 10–30, Tetra) pumping air through a 4-mm-diameter tube placed ∼4 mm away from the fly. A three-way solenoid valve (P/N AL4312, ASCO Scientific) driven by a USB 6210 DAQ card (National Instruments) controlled air flow. Wing sounds were picked up by a high-gain microphone placed ∼4 mm below the fly. Acoustic signals were digitized by a PC sound card controlled by a LabVIEW 8.6 script. The wing beat frequency (WBF) was determined using the short-time FFT procedure described in the study by Iyengar and Wu (2014).

Action potentials from the top-most dorsal longitudinal muscle (#45a; Miller, 1950) were recorded by an electrolytically sharpened tungsten electrode penetrating the flight muscle with a similarly constructed electrode inserted in the abdomen for reference. Signals were accessed with an AC amplifier (Microelectrode AC Amplifier, model 1800, A-M Systems; filter bandwidth, from 10 Hz to 20 kHz). Amplified signals were recorded with pulse code modulation (Neuro-Corder DR-484, Cygnus Technology) on videotape at a sampling rate of 44 kHz. Digitization of spiking traces [Fig. 1 (see also Fig. 6), Extended Data Fig. 5-1] was conducted by a digital acquisition card (USB 6210) controlled by custom-written LabVIEW scripts.

### Giant fiber pathway stimulation

As previously described (Engel and Wu, 1996), a pair of uninsulated tungsten electrodes were inserted in the eyes (anode in left eye) to pass stimulation of 0.1 ms duration (Isolated Pulse Stimulator, model 2100, A-M Systems). Long-latency response (LLR) and short-latency response (SLR) thresholds were first determined using increasing stimulation intensity with an interstimulus interval of 30 s. For the experiments involving LLR [twin-pulse refractory period (RP) and habituation], stimulus intensity was set at least 0.5 V below the SLR threshold. For SLR-related trials, stimulus intensity was set at 20 V, which was well above the SLR thresholds in all flies tested (for details, see Engel and Wu, 1992).

To determine the refractory period of LLR and SLR, twin-pulse stimuli were given every 10 s, with the stimulus interval decreasing stepwise (step size: LLR, 5–10 ms; SLR, 0.5–1 ms) from a starting interval (LLR, 100–800 ms; SLR, 10–20 ms). When a given interstimulus interval failed to trigger a second DLM spike in one or two trials, a 3–5 min rest was allowed before a train of six twin pulses of the same interstimulus interval were delivered at 0.1 Hz between twin pulses. If all six trials failed, then the previous stimulus interval was recorded as the refractory period.

The ability of GF to follow high-frequency stimulation was determined as described previously (Gorczyca and Hall, 1984; Engel and Wu, 1992). Three trains of 10 pulses at 200 Hz with 10 s in between were delivered, and the number of responses was counted.

### Habituation of GF-mediated LLRs and electroconvulsive stimulation-evoked seizures

For testing habituation rate, two trials of 100-pulse LLR stimulation trains at a given frequency (1, 2, and 5 Hz) were delivered with a 10 min interval between trials. The criterion for reaching habituation was the occurrence of the first five consecutive LLR failures (F5Fs; Engel and Wu, 1996). The larger F5F value of the two trials was recorded for the fly tested. Upon reaching habituation criterion, an air puff dishabituation stimulus was delivered to confirm habituation (for additional details, see Engel and Wu, 1996).

High-frequency electroconvulsive stimulation (0.1 ms pulses at 200 Hz for 2 s at a particular voltage of 15–80 V) were delivered across the brain to induce a stereotypic ECS discharge pattern (for details, see Lee and Wu, 2002). After high-frequency stimulation, test pulses of ≥20 V were sometimes delivered at 1 Hz to examine the failure and recovery of the GF pathway motor response. An interval of at least 10 min was allowed between each ECS to avoid the effect of refractoriness (Lee and Wu, 2002).

For follow-up experiments of DLM spike patterning during ECS discharges (see Fig. 6; Extended Data Fig. 5-1), spikes were detected and their timing recorded using custom-written MATLAB scripts (Iyengar and Wu, 2014; Lee et al., 2019). Firing rate was measured by the instantaneous firing frequency (ISI^−1^, defined as the reciprocal of the interspike interval, ISI between successive spikes), and firing regularity was quantified by the instantaneous coefficient of variation (CV_2_; Holt et al., 1996; Lee et al., 2019). Poincaré trajectories (PTs) were constructed by plotting the ISI^−1^ of a spike interval against the ISI^−1^ of the subsequent interval. Averaged ISI^−1^ versus CV_2_ plots were constructed as described in the study by Lee et al. (2019).

The neurophysiological properties of each fly were tested in the following sequence with 5 min of rest in between to minimize interference between protocols, as follows: flight, LLR and SLR thresholds, LLR refractory period, LLR habituation, SLR refractory period, the ability of SLR to follow high-frequency stimulation (30 pulses at 200 Hz), and ECS-evoked seizure discharges. Because of the weakness of aged flies and in some cases the absence of LLRs in the last 5% of survivors, not all of the flies went through every protocol. If the LLR was absent, all SLR-related protocols were still examined. Generally, all electrophysiological protocols took <1 h to complete for a single fly.

### Statistical analysis

For each age group tested (1, 5, 50, and 95% mortality), between 7 and 12 flies were generally tested. We have found that this sample size range is often sufficient to draw initial conclusions, based on our previous experiences studying ion channel and second-messenger system mutants (Engel and Wu, 1992, 1996; Lee and Wu, 2006; Iyengar and Wu, 2014). Individual flies were considered biological replicates, and all recorded data points were included in the analysis. Because of the non-normal distribution of datasets, the nonparametric Kruskal–Wallis ANOVA was used to determine cross-sectional differences between physiological parameters of flies from respective genotypes. The rank-sum test (with Bonferroni’s correction applied) was used as a *post hoc* test. Age-dependent trends in physiological parameters were identified by computing the nonparametric Spearman’s rank correlation coefficient (*r*_s_; Sokal and Rohlf, 1969). In the source data files accompanying each figure, the sample mean, median, SD, coefficient of variation, and the 25–75% tile interval are listed. To assess the robustness of the respective statistics, a bootstrap resampling approach (Efron, 1981) with 1000 replicates was used to estimate the 95th percentile confidence intervals. All statistical analyses were performed in MATLAB (r2019b).

## Results

### Flight performance and DLM firing activity across the life span: effects of high-temperature rearing and *Sod* mutation

The remarkable flight ability of *Drosophila* has been well documented (Heisenberg and Wolf, 1984; Dickinson, 2014), with flights often lasting for hours in tethered flies (Götz, 1968, 1987). To assess age-related changes in flight ability over the life span, we examined sustained tethered flight (Fig. 1*A*; Iyengar and Wu, 2014) to delineate characteristic alterations in flight muscle electrical activity patterns and corresponding biomechanical parameters, and to reveal the effects of high-temperature and oxidative stressors on the aging process.

As established in *Drosophila* and other organisms, the aging process manifests a nonlinear progression, indicated by varying rates of mortality at different stages in the life span (Fig. 1*B*; Vaupel et al., 1998; Extended Data Fig. 1-1, log-transformed mortality rate). We examined WT flies at 25°C and 29°C, two commonly used rearing temperatures in *Drosophila* aging studies. As previously reported, at 29°C the fly life span was nearly halved as compared with 25°C with a substantially accelerated mortality rate, convenient features frequently used to expedite aging studies (Fig. 1*B*; Loeb and Northrop, 1917; Leffelaar and Grigliatti, 1983; Ruan and Wu, 2008). At both temperatures, however, the life-span trajectories follow an inverse sigmoidal curve, with “plateau,” “shoulder,” and “tail” phases (Curtsinger et al., 1992). We have also determined the effects of increased oxidative stress by characterizing *Sod* mutant flies, reared at a slightly lowered rearing temperature, 23°C rather than 25°C to expand the rather short *Sod* life span for more precise chronological tracking of data. Notably, in the *Sod* life-span curve at 23°C, the early plateau phase is absent (Fig. 1*B*), and the slope of the mortality rate is negative across the life span (Extended Data Fig. 1-1). These distinctive features are previously reported *Sod* life span curves obtained at higher temperatures (25°C or 29°C; Phillips et al., 1989; Ruan and Wu, 2008), and highlight the characteristic high mortality of young *Sod* flies.

We found that flight ability was well preserved in some aged WT flies reared at either 25°C or 29°C, displaying robust tethered flights beyond the 30 s sustained flight duration, a cutoff criterion that effectively distinguishes flight-defective mutants from WT individuals (Iyengar and Wu, 2014). The “nonfliers” were mostly encountered in flies aged beyond the median life span (55 and 30 d for individuals reared at 25°C and 29°C, respectively; Fig. 1*C*). In striking contrast, many young *Sod* flies (∼50%) were not capable of sustained flight for >30 s, and this proportion further increased with age, with few *Sod* flies beyond the median life span (9 d) capable of flight for >30 s (Fig. 1*C*).

The differences between WT and *Sod* mutants in flight ability prompted us to examine the WBF as well as flight muscle DLM firing rate, across the life span. Using protocols established for the tethered flight preparation (Iyengar and Wu, 2014), we were able to acoustically monitor WBF via a microphone and simultaneously record DLM spiking activity (Fig. 1*A*,*D*). The WBF is largely determined by the natural mechanical resonance frequency of the thorax case, which is powered by alternating activation of two sets of indirect flight muscles, DLMs, and dorsal-ventral muscles or DVMs (Chan and Dickinson, 1996). Isometric contraction of indirect flight muscles is stretch activated and coincides with mechanical oscillation of the thorax, while their spiking activity, occurring at a much lower frequency than WBF, facilitates Ca^2+^ influx for force generation (Dickinson and Tu, 1997; Lehmann and Dickinson, 1997). Therefore, changes in muscle tension without shortening allow microelectrode recordings of the DLM spikes with minimal cell damage during prolonged flight.

Across the life span of WT flies, we noted a clear trend of increasing DLM firing rates, which nearly doubled in the oldest flies examined [Fig. 1*D*,*E*; Spearman rank correlation coefficient (*r*_s_) = 0.46, *p* = 9.9 × 10^−4^, and *r*_s_ = 0.58, *p* = 3.6 × 10^−4^, respectively, for 25°C-reared and 29°C-reared flies]. Accompanying this increasing trend, we noted an apparent overall increase in variability (Fig. 1*E*, dashed distribution outline). In contrast to this age-associated variability in DLM firing, the WBF showed little change throughout life span for the fliers in the three populations. In 25°C-reared WT flies, both young and old individuals displayed average WBF of ∼190 Hz, with a narrower spread within ±20 Hz (Fig. 1*F*). We observed only a small, but detectable, reduction in frequency across the life span of 29°C reared flies (*r*_s_ = −0.41, *p* = 0.19). Remarkably, the small population of *Sod* fliers displayed a largely normal DLM firing rate and WBF with an indicative upward trend in both DLM firing rate and WBF (*r*_s_ = 0.50, *p* = 0.06; and *r*_s_ = 0.60, *p* = 0.018, respectively; Fig. 1*E*,*F*). Together, our analysis of flight patterns suggests that the biomechanical properties of flight remained largely stable across the life span, while increases in DLM spiking mean frequency and variability reflected potential age-related alterations in central motor circuits.

In the following sections, we adopt the measure of “biological age” by tracking the percentage of mortality in the population to facilitate comparisons of the aging trajectories of functional alterations across different genotypes and conditions. This can be achieved by a renormalization of the time axis to reflect the cumulative mortality based on the chronology life-span curves (Fig. 1*B*). As shown in the following figures, we collected data at different biological ages, for WT flies reared at 25°C, biological ages at <1, 5, 50, and 95% mortality corresponded to the chronological ages of 7, 31, 50, and 72 d, whereas for 29°C rearing, this shifted to 7, 20, 30, and 35 d. Since *Sod* flies lacked a prolonged initial plateau phase but showed a lingering tail in the life-span curve, the biological ages targeted for data collection were modified to compress the first time interval to <5% mortality, and 70% mortality was added before the final 95% stage so as to allow sampling within the prolonged tail. The stages for *Sod* flies at <5, 50, 70, and 95% mortality therefore corresponded to 2, 9, 14, and 30 d. The distinct profile of *Sod* aging trajectory indicates markedly different effects exerted by oxidative and high-temperature stressors on the mortality rate along the life span. It is worth noting that despite the early precipitous drop and a shortened population medium life span, a small fraction of *Sod* mutant flies nevertheless exhibited relatively prolonged survivorship, giving rise to the characteristic, disproportionally prolonged tail in its life-span curve.

### Age-resilient and -vulnerable properties of the the GF circuit that mediates a jump-and-flight escape reflex

**Figure 2.**
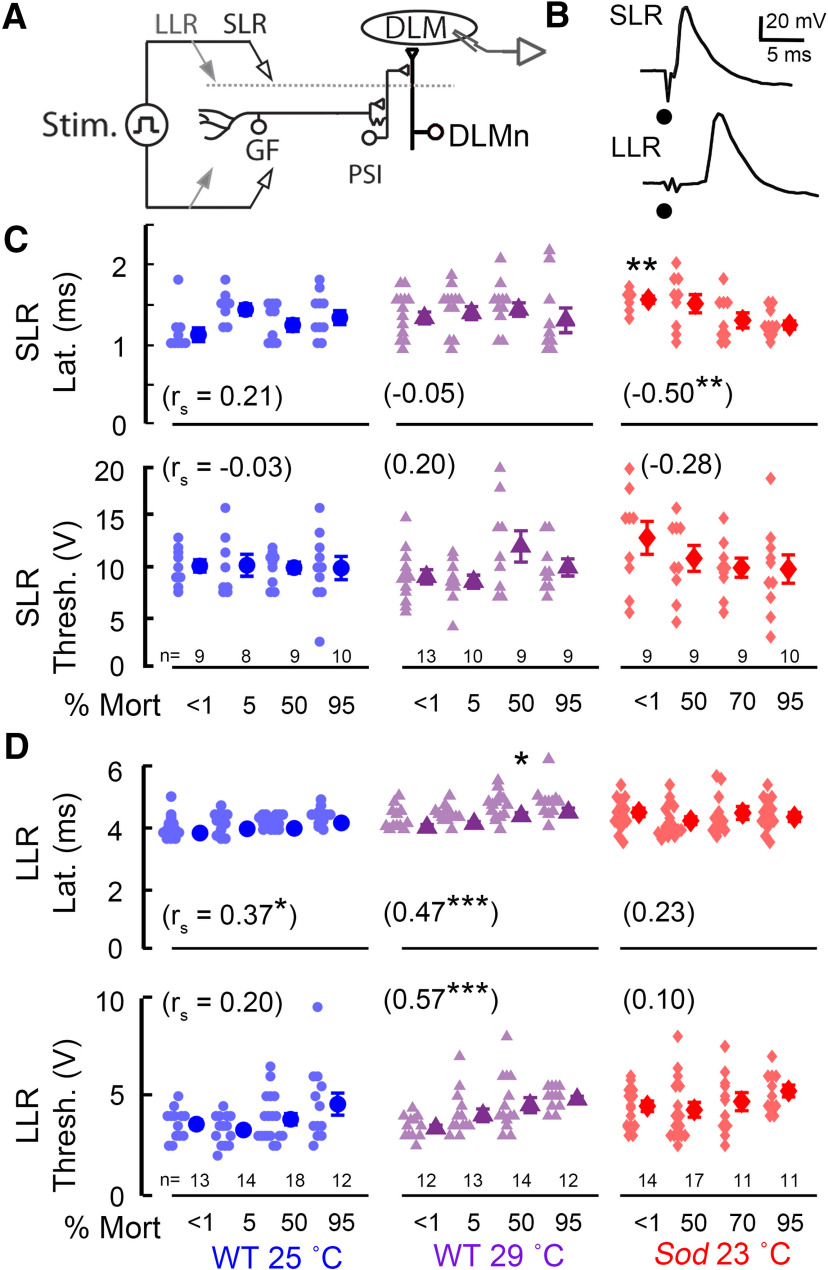
Performance characteristics of the GF pathway and its afferent inputs in aged WT and *Sod* mutant flies. ***A***, Overview of the GF “jump-and-flight” escape pathway. The GF neuron activates the indirect flight muscle DLM through a PSI that innervates the DLMn. For clarity, the GF branch that directly innervates the jump muscle (TTM) is not shown. ***B***, Electrical stimulation across the brain activates the GF pathway, and motor output is monitored via DLM action potentials in the tethered fly. High-intensity stimuli directly activate the GF neuron, giving rise to SLRs. Lower-intensity stimuli recruit GF afferent inputs, resulting in the DLM LLRs. Dots indicate stimulus artifact. ***C***, ***D***, SLR and LLR latency and threshold across the life span of WT and *Sod* flies. In this figure and in Figures 3 and 5, mean and SEM are indicated to the right of each dataset. Each lighter colored data point represents measurement from a single fly. Asterisks indicate significant difference against mortality-matched, 25°C-reared WT flies (Kruskal–Wallis ANOVA, rank-sum *post hoc* test). The numbers in parenthesis indicate Spearman correlation coefficient (r_s_) of the parameter measurements versus age categories. Stim., Stimulation; Mort, mortality; Lat., latency; Thresh., threshold.

One of the best-studied motor circuits in adult *Drosophila* is the GF pathway that triggers a jump-and-flight escape reflex critical for survival (Fig. 2*A*; Tanouye and Wyman, 1980; Trimarchi and Schneiderman, 1995b; von Reyn et al., 2017). Visual and other sensory inputs to the GF interneuron dendrites in the brain initiate the action potential propagating along the descending axon to drive a set of motor neurons in the thoracic ganglion. The DLM motor neuron (DLMn) receives the GF command signal indirectly via a peripherally synapsing interneuron (PSI) to evoke a DLM spike. This axoaxonal GF–PSI contact has been characterized as a mixed electrical and cholinergic synapse (King and Wyman, 1980; Gorczyca and Hall, 1984; Blagburn et al., 1999; Allen and Murphey, 2007). It has been well established that electric stimuli across the brain evoke two types of GF pathway-mediated DLM responses with distinct latencies (Fig. 2*B*). Low-intensity stimulation across the brain recruits afferent inputs to the GF neuron, thereby triggering a relatively LLR (∼4–6 ms; Elkins and Ganetzky, 1990; Engel and Wu, 1992, 1996), whereas high-intensity stimuli directly activate GF axonal spikes bypassing the brain afferents and resulting in an SLR (∼0.8–1.6 ms; Tanouye and Wyman, 1980; Engel and Wu, 1992).

We found clear distinctions between SLR and LLR in age-dependent modifications across the life span. Figure 2*C* shows robust maintenance of the basic properties (axonal conduction), of the GF neuron and downstream elements (PSI and DLM motor neurons) in aging WT flies, as indicated by the well preserved SLR parameters (Table 1, sample means, SDs, and coefficients of variation). No significant age-dependent alterations were observed in the latency or threshold of the SLR for either 25°C-reared or 29°C-reared WT flies, consistent with a previous report on well maintained SLR properties up to a late stage of the life span (Martinez et al., 2007). Nevertheless, we did observe a significant change in SLR properties in young *Sod* mutants, which displayed a retarded latency compared with 25°C-reared WT counterparts (mean SLR latency: 1.6 vs 1.1 ms; *p* = 0.002, Kruskal-Wallis ANOVA; Fig. 2*C*, Table 1). Furthermore, we noted an age-dependent decrease in this parameter for *Sod* (*r*_s_ = −0.50, *p* = 0.0015). This apparently paradoxical improvement brought the SLR latency parameters to a range directly comparable to those of WT flies, likely reflecting a progressive change in the *Sod* population composition, as healthier individuals persist and become better represented, while weaker ones die off.

**Table 1:** Features of GF pathway SLR and LLR in young and aged flies

	WT 25°C, % mortality				WT 29°C, % mortality				*Sod* 23°C, % mortality			
	<1	5	50	95	<1	5	50	95	<1	5	50	95
GF short-latency response (SLR) properties												
Threshold												
*N* (flies)	9	8	9	10	13	10	9	9	9	9	9	10
Mean (V)	10.1	10.2	9.9	9.9	9.0	8.6	12.1	10.0	12.9	10.8	9.9	9.8
SD (V)	1.9	3.1	1.7	3.7	2.6	2.1	4.7	2.5	5.0	3.9	2.9	4.5
CV	0.19	0.30	0.17	0.37	0.29	0.24	0.39	0.25	0.39	0.36	0.29	0.46
Latency												
*N* (flies)	9	8	9	10	13	12	11	9	10	9	9	10
Mean (ms)	1.1	1.5	1.3	1.4	1.3	1.4	1.4	1.3	1.6	1.5	1.3	1.2
SD (ms)	0.3	0.2	0.2	0.3	0.3	0.3	0.3	0.5	0.2	0.3	0.3	0.2
CV	0.27	0.13	0.15	0.21	0.23	0.21	0.21	0.38	0.13	0.20	0.23	0.17
Refractory Period												
*N* (flies)	9	8	9	10	10	9	9	8	9	7	9	10
Mean (ms)	3.8	4.6	4.3	5.1	3.8	4.7	6.0	7.1	9.2	4.1	3.7	5.3
SD (ms)	1.0	1.8	1.6	3.5	0.9	1.9	1.4	8.3	8.0	2.3	1.5	2.5
CV	0.26	0.39	0.37	0.69	0.24	0.40	0.23	1.17	0.87	0.56	0.41	0.47
Resp FF30												
*N* (flies)	9	8	9	10	10	8	9	8	9	8	8	10
Mean (# responses)	18.9	19.4	16.8	15.6	18.4	20.0	14.3	12.6	5.9	9.3	8.9	10.7
SD (# responses)	4.6	5.2	6.9	8.1	7.3	9.8	10.7	9.4	3.6	4.5	6.4	7.6
CV	0.24	0.27	0.41	0.52	0.40	0.49	0.75	0.75	0.61	0.48	0.72	0.71
GF long-latency response (LLR) properties												
Threshold												
*N* (flies)	13	14	18	12	12	13	14	12	14	17	11	11
Mean (V)	3.6	3.3	3.9	4.6	3.5	4.1	4.6	4.9	4.4	4.2	4.7	5.2
SD (V)	0.8	0.7	1.2	2.0	0.6	1.1	1.4	0.6	1.0	1.5	1.5	1.0
CV	0.22	0.21	0.31	0.43	0.17	0.27	0.30	0.12	0.23	0.36	0.32	0.19
Latency												
*N* (flies)	13	14	18	12	12	13	14	12	14	17	11	11
Mean (ms)	4.1	4.3	4.3	4.5	4.3	4.4	4.7	4.8	4.5	4.2	4.4	4.3
SD (ms)	0.4	0.3	0.2	0.3	0.4	0.3	0.4	0.5	0.5	0.5	0.7	0.5
CV	0.10	0.07	0.05	0.07	0.09	0.07	0.09	0.10	0.11	0.12	0.16	0.12
Refractory Period												
*N* (flies)	13	14	18	12	12	13	14	12	14	17	11	11
Mean (ms)	41.2	45.4	38.3	87.9	67.5	49.2	145.9	162.5	106.1	71.5	89.0	120.9
SD (ms)	22.2	43.5	18.5	90.8	35.6	23.9	123.5	165.9	68.1	24.9	66.6	66.3
CV	0.54	0.96	0.48	1.03	0.53	0.49	0.85	1.02	0.64	0.35	0.75	0.55

In contrast to the robust SLR properties, LLR parameters revealed clear changes in afferent inputs from higher centers to the GF neuron. Strikingly, among the oldest WT flies (e.g., 25°C, 95% mortality), LLRs were not elicited in more than half of them (14 of 26), indicating severely compromised afferent inputs to the GF. Additionally, both 25°C-reared and 29°C-reared WT populations displayed a small (<10%), but detectable, age-dependent increases in LLR latency (*r*_s_ = 0.37 and 0.47; *p* = 0.003 and 0.0005, respectively; see also Table 1), and a modest age-dependent increase in LLR threshold (*r*_s_ = 0.57, *p* = 1.3 × 10^−5^) was found in 29°C-reared flies. These relatively stable LLR properties were not grossly disrupted in the *Sod* mutant flies that remained responsive (Fig. 2*D*).

In summary, our SLR results indicate that the GF and its downstream elements, including the PSI and DLM motor neurons, remain robust and could reliably transmit vital signals to evoke an escape reflex even in aged flies. However, the input elements upstream of the GF neuron showed more clear age-dependent functional decline, which could lead to a catastrophic collapse of LLR in some of the extremely old individuals (95% mortality) that were devoid of LLR.

### Alteration of SLR and LLR refractory period by high-temperature and oxidative stressors

The robust, well maintained basic properties of the GF and its downstream elements provide reliable readouts for examination of activity-dependent plasticity across the life span to uncover aging-vulnerable neural elements upstream of the GF neuron. By using well established stimulus protocols in the tethered fly preparation for activity-dependent processes, including paired-pulse refractory period and habituation to repetitive stimulation (Gorczyca and Hall, 1984; Engel and Wu, 1996), we were able to establish modification because of aging in these use-dependent properties, as well as their alterations by high-temperature or oxidative stressors.

**Figure 3.**
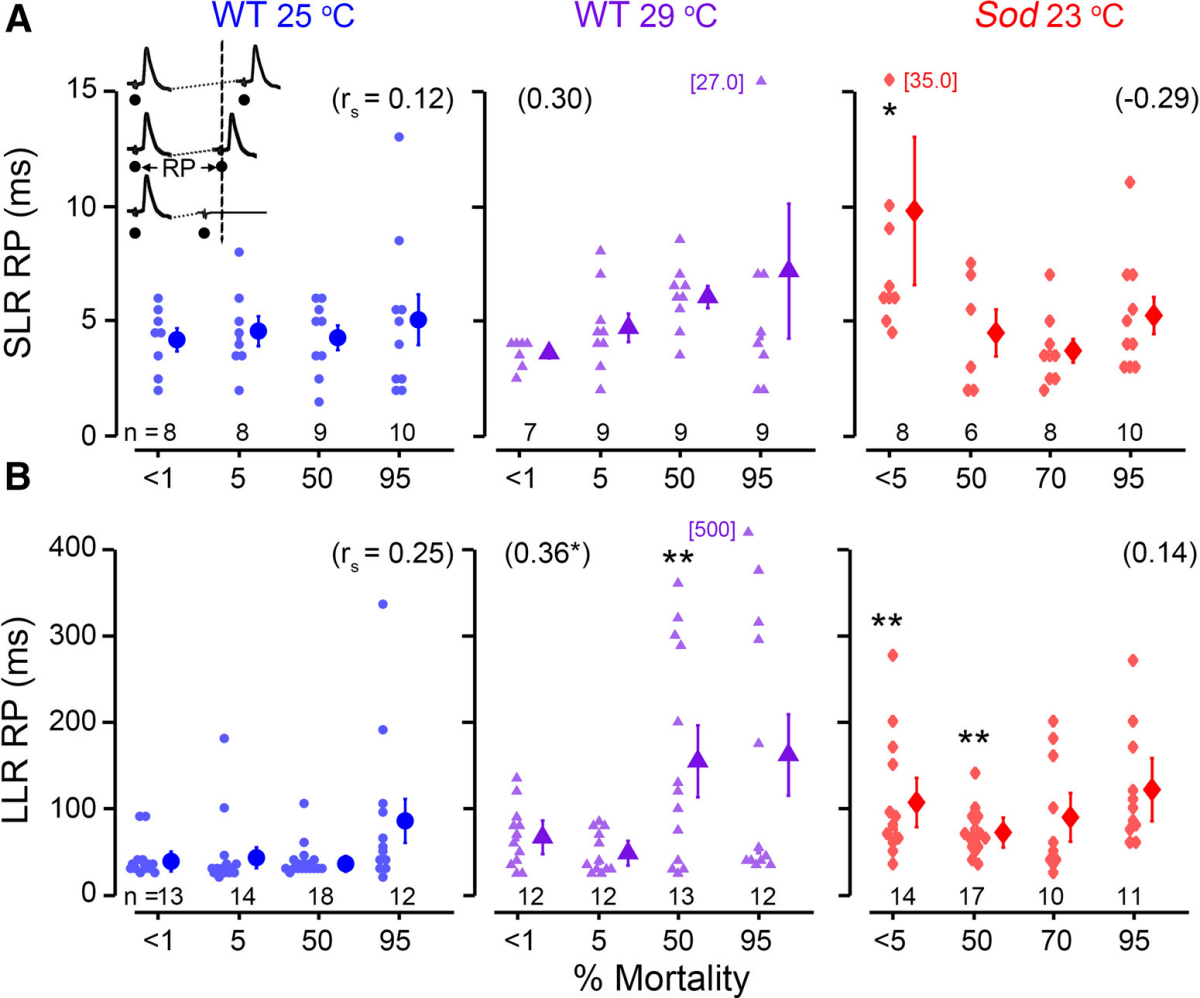
Paired-pulse refractory characteristics of GF-mediated DLM short- and long-latency responses. The paired-pulse RP represents the shortest interstimulus interval where the second stimulus evokes a DLM spike; shorter stimulus intervals fail to recruit two spikes (inset). ***A***, ***B***, Scatter plots of the SLR (***A***) and LLR (***B***) RPs from WT flies reared at 25°C and 29°C as well as *Sod* flies reared at 23°C are shown over the course of their life spans, presented as a percentage mortality. Outlier values exceeding the axis range are indicated adjacently. See the legend of Figure 2 for statistical treatment and presentation. Data from the related SLR following frequency (i.e., FF_30_) protocol is shown in Extended Data Figure 3-1.

The RP, for both SLR and LLRs, is defined as the shortest interval between paired stimulus pulses at which a second DLM response could be evoked (Fig. 3*A*, inset). We found WT flies across the life span displayed similar SLR RP performance when reared at 25°C, while 29°C-reared WT flies displayed an apparent, but not statistically significant trend of increasing RP over the life span. In a complimentary set of experiments, we delivered 30 SLR-evoking stimuli at 200 Hz and recorded the number of successful responses (following frequency, FF_30_). Across the life span, both 25°C-reared and 29°C-reared flies displayed small alterations in performance, with changes not reaching statistical significance (Extended Data Fig. 3-1). The refractoriness of LLR in older WT flies reared at 25°C was similar their younger counterparts. However, 29°C-reared WT flies showed a significant age-dependent increase in LLR RP, most evident in the older populations (Fig. 3*B*; 50 and 95% mortality). Notably, the distributions of SLR and LLR RPs in aged groups showed larger skews, with several individuals displaying disproportionally lengthened refractory periods (less pronounced in SLR RP but most evident in LLR RP; Fig. 3*B*). Furthermore, the oldest groups (95% mortality) consistently displayed the largest coefficients of variation in LLR refractory period datasets (Table 1). These observations highlight the stochastic nature of age-related functional deterioration in the upstream circuit elements afferent to the GF pathway.

10.1523/ENEURO.0443-21.2021.f3-1 Figure 3-1 Giant fiber SLR properties across the life span: FF_30_ protocol. The FF_30_ protocol measures the ability of the GF pathway to follow high-frequency stimulation. Three trains of 10 stimuli are delivered at 200 Hz with a 10 s interval between trains (30 total stimuli). The number of responses was recorded (Resp 30 P), with a higher response rate corresponding to a better ability to follow high-frequency stimulation. Sample sizes as indicated for each age group, and the *r*_s_ is shown above. No population displayed statistical significance age-dependent trend (i.e., *p* > 0.05). Download Figure 3-1, TIF file.

Remarkably, young *Sod* flies represented the most heterogeneous population among different age groups in terms of refractory periods. Presumably, the population included a portion of severely defective, very short-lived individuals that accounted for the initial drop and the diminished plateau phase of the life-span curve (Fig. 1*B*). Our observations revealed significant lengthening in both SLR and LLR refractory periods in very young (<5% mortality) *Sod* mutants (SLRs, *p* < 0.05; LLRs, *p* < 0.01; Kruskal–Wallis ANOVA, Bonferroni-corrected rank-sum *post hoc* test; Fig. 3*A*,*B*). Similarly, *Sod* mutants displayed significantly lower FF_30_, indicating worse performance compared with WT counterparts (Extended Data Fig. 3-1). In conjunction with a clear increase in SLR latency (Fig. 2*C*), the RP lengthening point toward severe GF defects in a subpopulation of young *Sod* flies. On the other hand, the surviving aged *Sod* mutants showed relatively stable SLRs and LLRs, with less substantial age dependence at >50% mortality (Fig. 3*A*,*B*).

### Aging progression of higher-level activity-dependent signal processing: acceleration of LLR habituation over life span

The above results suggest the functional integrity of the GF and its downstream elements as indicated by SLR performance (Figs. 2, 3) is age resilient across the life span. Nevertheless, high-temperature stress and particularly *Sod* mutation can exert clear effects on activity-dependent aspects of GF-mediated DLM responses, as indicated by refractory period of LLRs (Fig. 3*B*). In *Drosophila*, the jump-and-flight startle reflex shows habituation on repetitive visual stimulation (Trimarchi and Schneiderman, 1995a), which is recapitulated in the habituation of LLRs (Engel and Wu, 1996). Habituation represents a nonassociative form of learning that involves experience-dependent plasticity in higher functions (Engel and Wu, 2009; Rankin et al., 2009).

**Figure 4.**
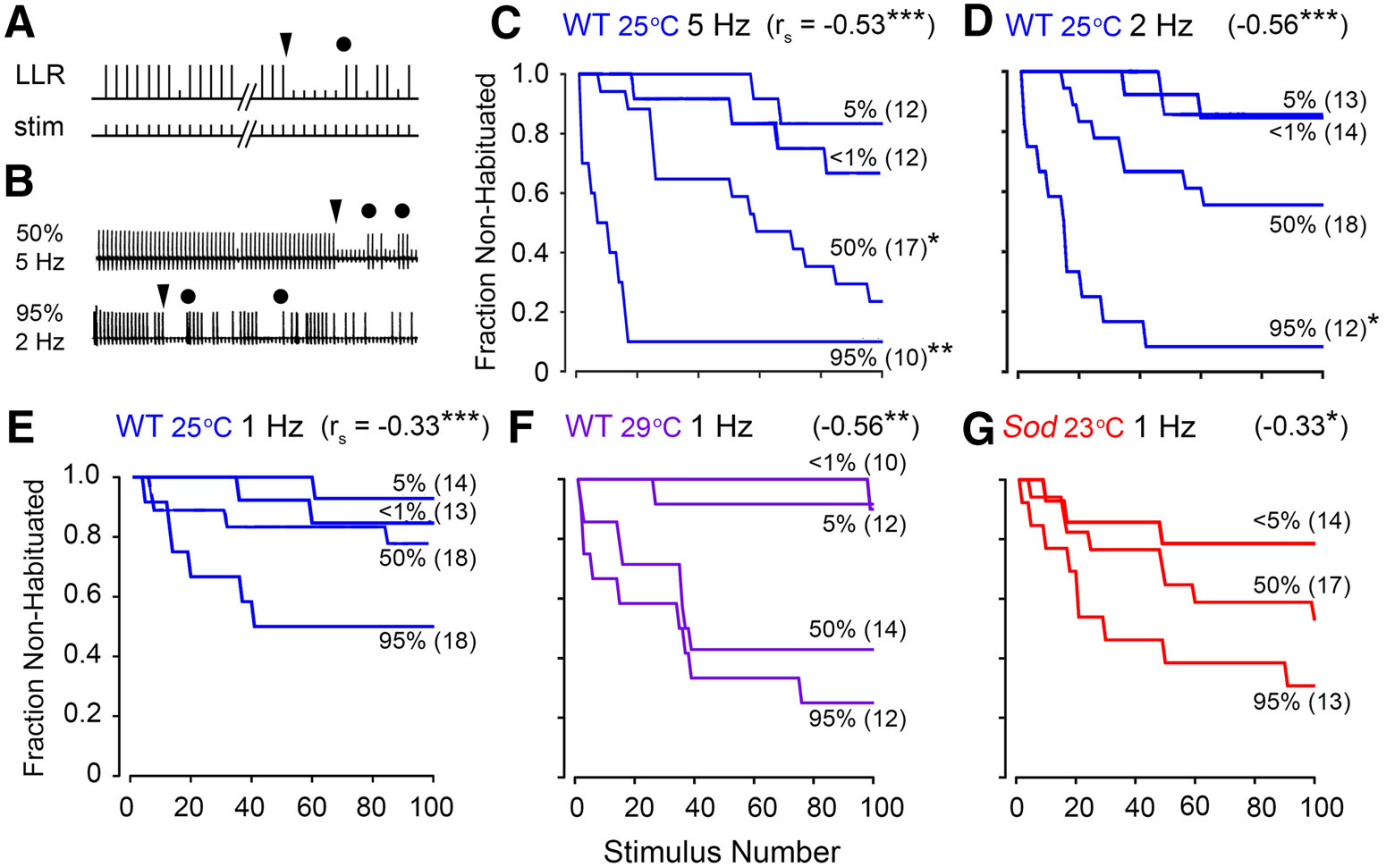
Age-dependent alterations in habituation kinetics of GF afferents. ***A***, Habituation of GF afferents. Habituation is operationally defined as the first instant of reaching five consecutive DLM spike failures to LLR-evoking stimulation train of defined frequency (triangle; Engel and Wu, 1996). Subsequent dishabituation stimulus (circle, air puff) triggered recovery of LLRs, excluding sensory adaptation or motor fatigue as the basis for DLM response failure. ***B***, Sample traces from WT flies reared at 25°C of slower habituation at 50% mortality (top trace, 5 Hz stimulation) and faster habituation at 95% mortality (lower trace, 2 Hz stimulation). ***C–E***, Frequency dependence of LLR habituation in 25°C-reared WT flies to stimulation at 5 Hz (***C***), 2 Hz (***D***), and 1 Hz (***E***). The fraction of flies in the sample population of each age group (<1, 5, 50, and 95% mortality) that were not habituated after a given number of stimuli is plotted. ***F***, ***G***, Habituation in WT 29°C-reared flies (***F***) and in *Sod* mutants (***G***) to 1 Hz stimulation. Sample sizes are as indicated next to each plot. Asterisks at the end of the trace indicate significantly faster habituation compared with habituation in mortality-matched WT 25°C-reared flies to 1 Hz stimulation (log-rank test). The age-dependent *r*_s_ is indicated in the top right corner of each panel.

To further investigate age-related changes in the plasticity of upstream processing of GF inputs, we examined habituation of LLRs (Engel and Wu, 1996, 1998). Upon evoking LLRs repetitively, the DLM responses habituate, as subsequent stimuli fail to recruit LLRs (Fig. 4*A*). However, LLRs may be rapidly restored following the application of a dishabituation stimulus of a different modality, such as an air puff, excluding sensory adaptation or motor fatigue as the basis for LLR failure (Fig. 4*A*). We applied 100 LLR-evoking stimuli at three frequencies (1, 2, and 5 Hz) and adopted a habituation criterion as the number of stimuli required to reach five consecutive LLR failures (F5Fs; Fig. 4*A*,*B*; Engel and Wu, 1996). The large number of stimuli used in this protocol provides greater power to resolve age-dependent alterations in an intrinsically stochastic process.

Across the three stimulation frequencies examined (1, 2, and 5 Hz) for WT flies reared at 25°C, we found that between <1% and 5% mortality, during the plateau portion of the life span, the LLR habituation rate was largely unaltered, with a large fraction of flies showing little habituation throughout the stimulation train (Fig. 4*C–E*). However, during the mortality phase of the life-span curve, ≥50% mortality, we observed a marked acceleration in LLR habituation, with the oldest flies habituating the fastest (*r*_s_ between −0.33 and −0.56, *p* < 0.001). As expected, we found more evident acceleration in habituation rate at higher stimulation frequencies (Fig. 4*C–E*), which was most pronounced in the older populations (50 and 95% mortality) examined. Together, our findings suggest that the brain circuits afferent to the GF pathway remained largely unaltered during the plateau portion of the life-span curve, a substantial fraction of the life span. Nevertheless, across the life span, we could delineate the distinct habituation rates associated with the 5, 50, and 95% age groups, particularly at the higher simulation frequency of 5 Hz, offering a quantifiable index of aging progression.

High-temperature rearing and *Sod* mutations further accelerated habituation. Compared with 25°C-reared counterparts, 29°C-reared WT flies displayed considerably faster habituation to 1 Hz stimulation in aged groups (50 and 95% mortality; compare Fig. 4, *E*, *F*). Furthermore, *Sod* mutants (at 23°C) showed an accelerated habituation rates compared with WT counterparts even in younger flies (<5% mortality; compare Fig. 4, *G* and *E*, *F*), presumably correlated with the pronounced increase in LLR paired pulse refractory period in this age group (Fig. 3*B*). Therefore, among the temperature- or oxidation-stressed groups, the habituation rate again can serve as a physiological marker of age progression even at low frequencies (∼1 Hz) of repetitive stimulation.

### Increasing susceptibility to electroconvulsive induction of seizures across the life span

**Figure 5.**
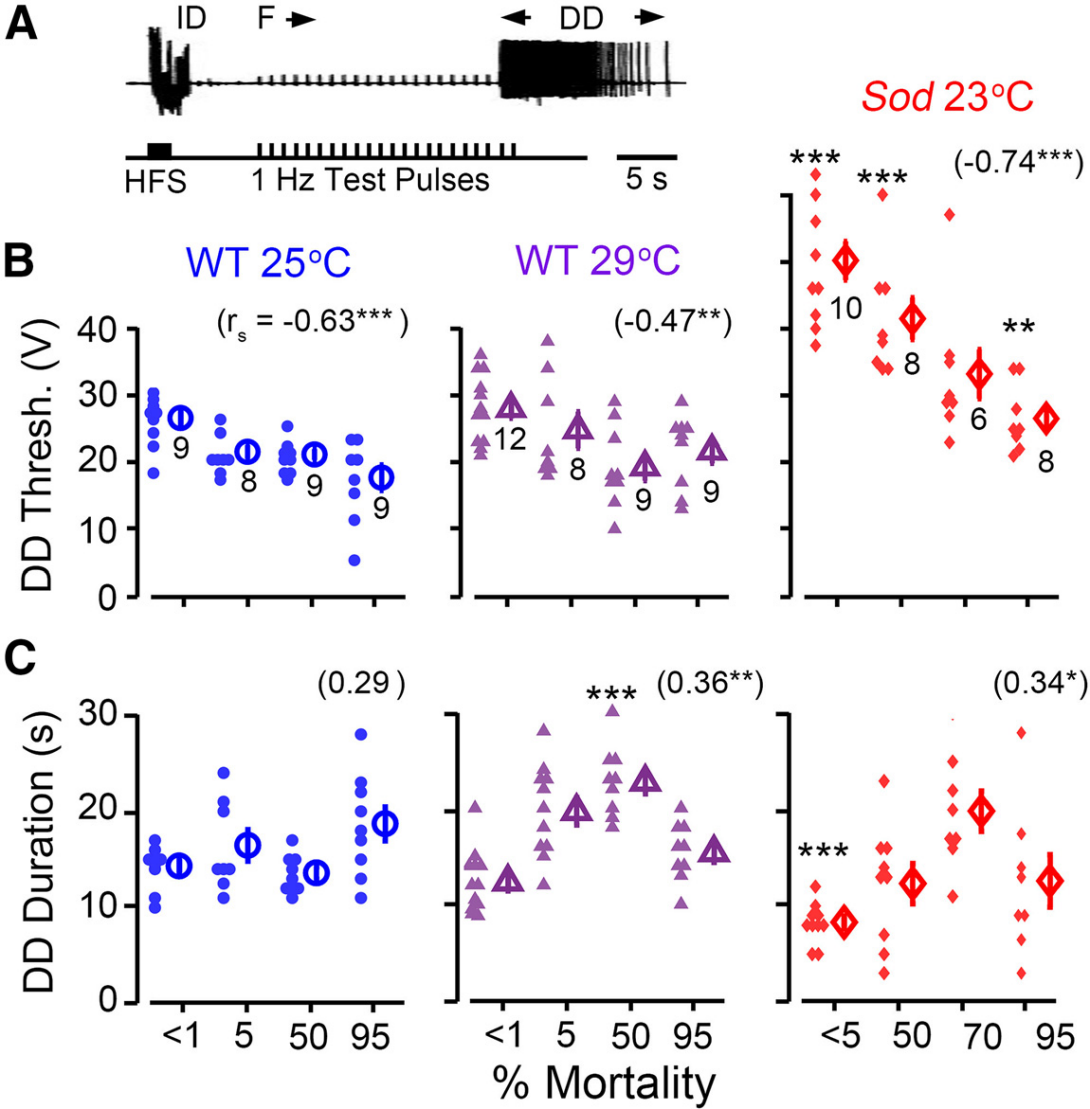
Decreasing threshold of ECS discharges in aging flies. ***A***, ECS activity was evoked by a short train (2 s) of high-intensity, high-frequency stimulation (HFS; 0.1 ms pulse duration, 200 Hz) across the head in tethered flies. DLM spike discharges serve as a convenient monitor of these seizures, and the pattern of these discharges is highly stereotypic (Lee and Wu, 2002), consisting of an spike ID, followed by a period of paralysis corresponding to failure of test pulse-evoked GF pathway responses (***F***), and a spike DD. The example trace here shows the sequence in a young 25°C-reared WT fly. ***B***, ***C***, Scatterplots of the stimulation threshold to induce DD (***B***), and of the duration of the evoked DD (***C***) across the life spans of 25°C-reared and 29°C-reared WT flies and *Sod* mutants. Mean and SEM are indicated to the right of each group, with sample sizes below. See the legend of Figure 2 for other details of statistical treatment and presentation (Extended Data Figure 5-1, analysis of spiking during the DD).

In addition to acting as an output of the flight pattern generator and GF pathway-mediated escape reflex described above, DLM action potentials may serve as reliable indicators of CNS spike discharges associated with seizures (Pavlidis and Tanouye, 1995; Lee and Wu, 2002). Such seizure episodes may reflect aberrant spiking originating from motor pattern generators located in the thorax, such as flight (Harcombe and Wyman, 1977; Iyengar and Wu, 2014) and grooming (Engel and Wu, 1992; Lee et al., 2019). As shown in Figure 5*A*, high-frequency stimulation across the brain (200 Hz, 2 s train of 0.1 ms pulses; Lee and Wu, 2002) can trigger a highly stereotypic ECS discharge repertoire, consisting of an initial discharge (ID), a period of paralysis reflected by GF pathway failure (F; in response to 1 Hz test pulses), and a second delayed discharge (DD) of spikes. This experimental protocol provides several stable and quantitative measures, within individual flies, suitable for assessing age-related modifications of CNS function, including the threshold to induce seizures (by adjusting intensity from 15 to 80 V; Fig. 5*B*) and the discharge duration of DD spike trains (Fig. 5*C*).

10.1523/ENEURO.0443-21.2021.f5-1 Figure 5-1 DD spiking across the life span. ***A***, Example traces of DD spiking in young (top row) and aged (bottom row) flies. ***B***, Scatterplot of the spike count during the DD. Trend line indicates the Gaussian kernel running average as computed in Figure 1. The sample size and age-dependent *r*_s_ are indicated above (*p < 0.05, ***p < 0.001). Download Figure 5-1, TIF file.

We found a clear trend of increasing seizure susceptibility with age in WT and *Sod* flies (Fig. 5*B*). WT flies reared either at 25°C or 29°C displayed a substantial reduction in the DD threshold across their life span (−33% and −23%, respectively, for <1% vs 95% mortality). In *Sod* mutants, the trend was even more pronounced (−46% for 5% vs 95% mortality). The effects of the *Sod* mutation were most striking in the youngest age group. In these flies (<5% mortality), the threshold was significantly higher than WT counterparts (*p* < 0.001, Kruskal–Wallis ANOVA, rank-sum *post hoc*), corroborating the other observations of extreme physiological defects found in this age group (Figs. 2, 3). Furthermore, this increase in ECS threshold was present across the *Sod* life span (*p* < 0.05, Kruskal–Wallis ANOVA, rank-sum *post hoc*), suggesting that elevated oxidative stress could render seizure-prone circuits less excitable.

Despite individual variability within each fly group, significant correlation coefficients suggest an enhanced excitability of seizure-prone circuits in aged flies (Fig. 5*B*). Compared with other potential indicators (e.g., habituation rate; Fig. 4), this age-dependent monotonic decrease of ECS threshold could provide a tighter, more quantitative measure of aging progression.

In contrast to the decreasing trend of seizure threshold over the life span, there was a milder trend of increasing DD duration in WT flies reared at 29°C or in *Sod* mutants, but ending with a substantial drop in the oldest (95%) populations (Fig. 5*C*; WT 29°C-reared flies: *r*_s_ = 0.36, *p* = 0.0041; *Sod* flies: *r*_s_ = 0.34, *p* = 0.047). However, there was no clear age-dependent trend of increasing DD duration in 25°C-reared WT flies (Fig. 5*C*). In a complementary dataset acquired at higher temporal resolution to resolve individual spikes, we examined the total number of spikes during the DD in these populations. Mirroring the DD duration observations, we found that 25°C-reared WT flies did not display an age-dependent trend, while WT flies reared at 29°C, as well as *Sod* mutants, showed modest increases in the DD spike count (Extended Data Fig. 5-1; WT 29°C-reared flies: *r*_s_ = 0.50, *p* = 1.4 × 10^−5^; *Sod* mutants: *r*_s_ = 0.27, *p* = 0.04).

### Age trajectories of ID firing patterns reveal a potential predictor of mortality rate

**Figure 6.**
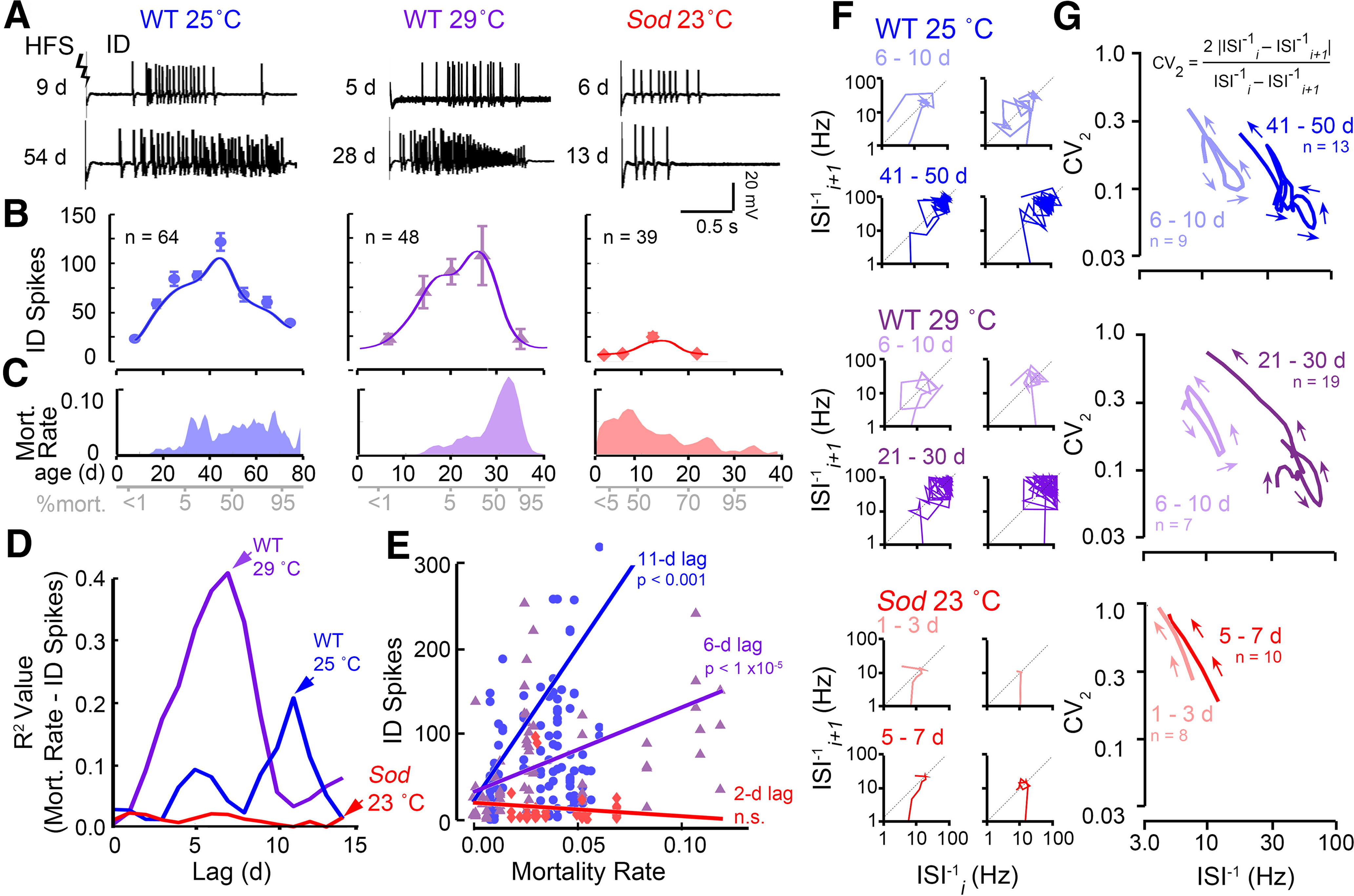
Age-dependent alterations in ID spike counts versus mortality rates and signatures of ID spiking trajectories in WT and *Sod* flies. ***A***, Representative high-frequency stimulation-induced IDs from young (<1% mortality: 9, 5 and 6 d traces) and aged (50% mortality: 54, 28 and 13 d traces) WT and *Sod* flies (stimulation voltage, 80 V). ***B***, Plot of the number of spikes within the ID across the life span (mean ± SEM for different age groups, and sample sizes across the populations are indicated). Trend lines represent a Gaussian kernel running average (Fig. 1) of the age trajectory for the three populations. ***C***, Plot of the mortality rates (defined as the negative slope of the life-span curve (Fig. 1*B*) across the life span for the three populations examined (Extended Data Figure 1-1, log-transformed mortality rate plots). ***D***, Linear cross-correlation between the mortality rate and ID spike count on introducing a lag of 0–15 d to determine the optimal lag for maximal correlation (i.e., 11 and 6 d for 25°C-reared and 29°C-reared WT flies). In *Sod* mutants, no significant correlation between mortality rate and ID spike count was detected. ***E***, Plot of best correlation between of lagged ID spike count and mortality rate for the three populations (*p* values are as indicated previously). ***F***, Poincaré plots of representative IDs from young (<1% mortality) and aged (∼50% mortality) flies. The instantaneous firing frequency of each ISI^−1^*_i_* is plotted against the ISI^−1^*_i+_*_1_ on a log scale (see text). ***G***, Averaged trajectories (as constructed in Lee et al., 2019) of the ISI^−1^ versus the CV_2 _ (as defined by Holt et al., 1996; see text). Lower values of CV_2_ indicate more rhythmic firing. The number of trajectories used to construct the average is as indicated. Note the similar changes in aged 25°C-reared and 29°C-reared WT flies compared with young counterparts and distinctions between WT and *Sod* mutant discharges.

Alterations in ECS threshold and DD duration across life span (Fig. 5) call for detailed characterization of ID spiking to extract additional salient features associated with aging progression. Since ID occurs as a brief burst (1–4 s; Figs. 5*A*, 6*A*) of high-frequency spiking activity, a set of ECS data at a higher temporal resolution enabled an analysis of ID spiking dynamics at the level of individual spikes (Fig. 6*A*). Immediately following electroconvulsive stimulation, the ID spike patterns manifested striking, but more complex, age dependence and vulnerability to oxidative stress. In younger WT flies (reared at either 25°C or 29°C), ID spike counts progressively increased with age until ∼50% mortality (Fig. 6*A*,*B*). Beyond 50% mortality, a decline in the ID spike count became apparent; notably, the oldest flies (95% mortality) regressed to a spike count similar to those of young flies (<1% mortality). In contrast to the nearly 10-fold range for ID spike counts across the WT life span, *Sod* mutants displayed only rather sparse spiking, too few to shape a prominent age-related profile in their counts (Fig. 6*A*,*B*). This observation mirrors the particular vulnerability of ECS discharges to oxidative stress, along with the drastically elevated threshold levels in ECS induction in *Sod* mutant flies (Fig. 5*B*).

We noted that the curve of the average ID spike count across the life span showed a gross resemblance in overall profile with the mortality rate curve. The progression of spike count changes apparently preceded the mortality rate changes in WT (compare Fig. 6, *B*, *C*). We sought an appropriate temporal shift between the ID spike count and mortality rate curves that would optimize regression fit. The iterations yielded the best correlation between the ID spike count and mortality rate with 11 and 6 d lags for WT flies reared at 25°C and 29°C, respectively (Fig. 6*D*,*E*). These observations suggest that the ID spike production over the life span may serve a predictor for the ensuing mortality rate in WT flies. In other words, along with other physiological indicators for aging progression examined above (Figs. 4, 5*B*), ID spike counts might also provide a gross forecast of the mortality rate, which is the derivative of the life-span curve. However, this relationship may not be applicable to a variety of genotypes since it does not seem to hold true for *Sod* flies (Fig. 6*A*,*B*).

### Signatures of ID spiking patterns in young and old WT and *Sod* flies

Salient features of spike patterns can be uncovered by systematical tracking of the successive changes in consecutive spike intervals to depict the spike train dynamics, as shown in the Poincaré plots (Fig. 6*F*; Lee et al., 2019). In these plots, instantaneous firing frequency, defined as the inverse of an inter-spike interval (ISI^−1^), was first determined for each of the successive spike intervals in a spike train. Each ISI^−1^ was then plotted against the ISI^−1^ of the following spike interval, and the process was reiterated sequentially for each spike in the ID spike train (i.e., ISI^−1^*_i_* vs ISI^−1^*_i+_*_1_ for the *i*th spike in the sequence). For strictly periodic spiking, all data points of the PT would fall at a single location along the diagonal. Irregular deviations from rhythmicity between successive spike intervals appear as perpendicular shifts away from the diagonal. Thus, the trajectory depicts the evolution of spiking frequency, while excursion patterns about the diagonal in the PT indicate characteristics of recurrent firing patterns. Poincaré plots of representative individual spike trains are shown in Figure 6*F*, which readily capture the larger number of spikes with increasing ISI^−1^ values in aged WT flies compared with younger counterparts (up to 100 vs <30 Hz). In contrast, the few ID spikes in *Sod* flies, regardless of age, occurred in a lower frequency range (∼10 Hz).

To integrate information embedded in individual PTs to indicate overall differences in ID spiking dynamics between age groups, we replotted the PTs to carry both frequency and local variation for successive ISIs to combine them into an “average” trajectory (Fig. 6*G*; see Materials and Methods; Lee et al., 2019, computational details). Briefly, for each ISI in the ID spike sequence, the ISI^−1^ was plotted against the CV_2_, defined as follows: 2 | ISI^−1^*_i_* – ISI^−1^*_i+_*_1_|/[ISI^−1^*_i_* + ISI^−1^*_i+_*_1_] (Holt et al., 1996). Along the trajectory, low CV_2_ values correspond to more regular firing (lower fractional changes among adjacent spike intervals), while high CV_2_ values indicate where more abrupt changes in spike intervals occur. The IDs of young WT flies (<1% mortality) reared at either 25°C or 29°C displayed rather similar ISI^−1^ versus CV_2_ trajectories compared with each other, with peak ISI^−1^ between 10 and 20 Hz and a similar range of CV_2_ in the spiking procession. Remarkably, for both rearing temperatures, the trajectories of aged WT flies displayed similar transformation from their young counterparts, with more complex, right-shifted trajectories with peak ISI^−1^ s > 70 Hz (Fig. 6*G*; 41–50 and 21–30 d, respectively, for 25°C and 29°C rearing), suggesting that the changes in circuit activity patterns across the life span are conserved during high-temperature rearing. Nevertheless, the simple ISI^−1^ versus CV_2_ trajectories of both young and aged *Sod* stood in sharp contrast to WT trajectories, providing another major distinction between the effects of high-temperature and oxidative stressors and underscoring the hypoexcitable nature of the *Sod* mutant.

Clearly, these “average” trajectories present concisely the overall characteristics of the ID spiking activity, with precise temporal information from the initiation to cessation of the spike train. They are effective signatures for IDs of different age groups in each genotype; WT flies reared at 25°C and 29°C traverse similar terrains in the span of ID spike trains, whereas *Sod* flies of various ages yield only abbreviated signatures, marking very limited terrains in specific regions in the frequency-variance landscape.

## Discussion

This report summarizes our initial efforts toward a quantitative description of neurophysiological changes during aging in several well established motor circuits in *Drosophila*. Aging studies in flies have largely focused on how the life span is influenced by genetic modifications in cellular biochemical pathways, such as those regulated by insulin-like peptide (Clancy et al., 2001; Hwangbo et al., 2004) and target of rapamycin (Kapahi et al., 2004) signaling and oxidative stress (Tower, 1996; Phillips et al., 2000), as well as by environmental factors, including diet (Mair et al., 2003; Piper and Partridge, 2007; Skorupa et al., 2008) mating experience (Kuo et al., 2012; Gendron et al., 2014; Harvanek et al., 2017), exercise (Piazza et al., 2009; Sujkowski et al., 2015), and trauma (Katzenberger et al., 2013, 2015). In parallel, sev eral established behavioral assays have provided readouts of age-related functional decline, including fast phototaxis and negative geotaxis (Miquel et al., 1976; Arking and Wells, 1990; Simon et al., 2006), flight (Miller et al., 2008), memory (Tamura et al., 2003; Yamazaki et al., 2007), courtship (Cook and Cook, 1975; Partridge et al., 1987), as well as sleep and other circadian activity patterns (Koh et al., 2006; Bushey et al., 2010). However, behavior-relevant studies registering concomitant changes in neurophysiological processes in *Drosophila* have been largely limited to electroretinogram (Ueda et al., 2018), peripheral neuromuscular synapses (Banerjee et al., 2021), or GF pathway SLR properties (Martinez et al., 2007; Augustin et al., 2018, 2019; Blagburn, 2020). Our work provides an initial glimpse across an array of motor circuits including the upstream processing of the GF-mediated escape circuit, and pattern generators driving flight and seizure discharges. Furthermore, our observations provide a first-order quantitative comparison of age trajectories, revealing drastically different effects of high-temperature rearing and oxidative stress on the aging progression of various neurophysiological parameters in WT and mutant flies.

### Aging-resilient and -vulnerable aspects of motor circuit function in *Drosophila*

**Figure 7.**
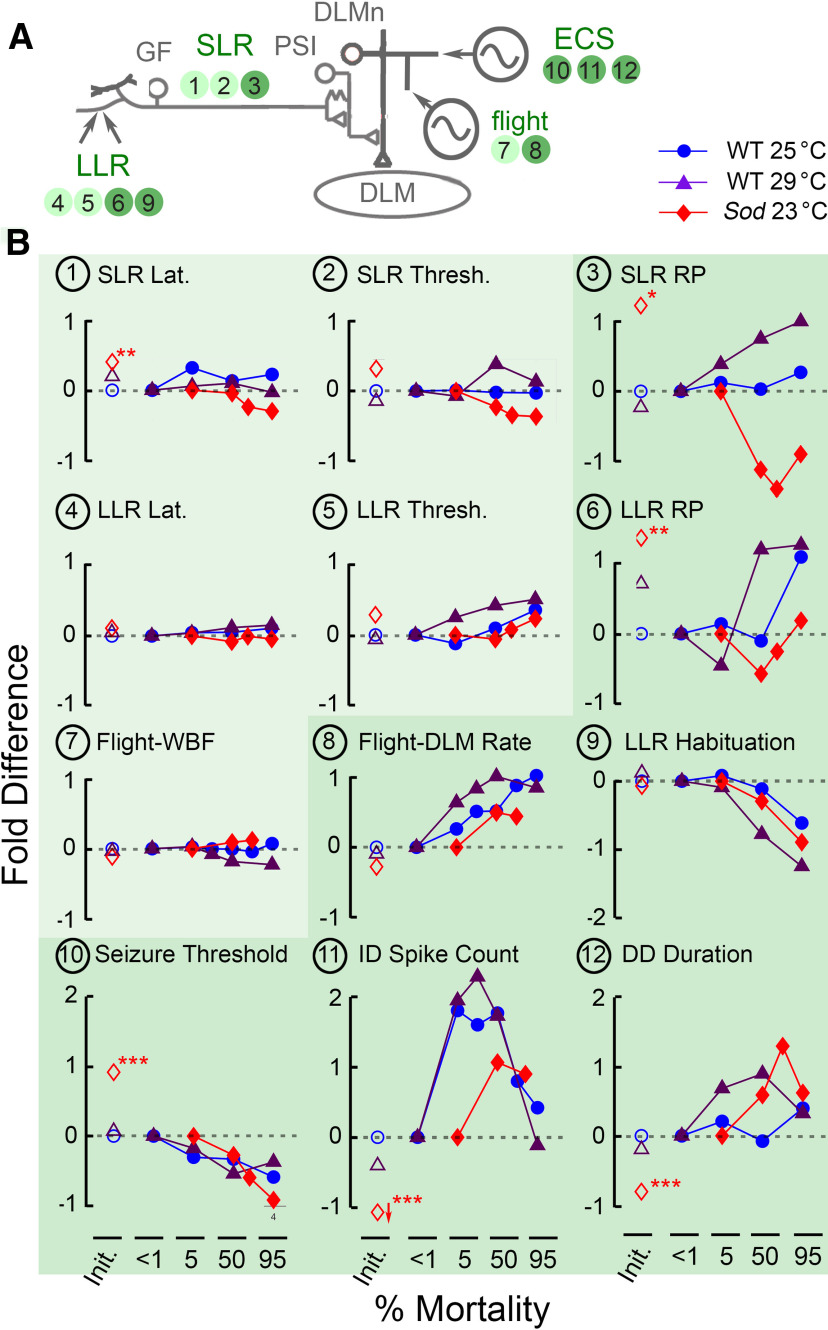
Distinct age trajectories of different motor circuit properties examined in this study. ***A***, Schematic diagram of the circuit components generating motor patterns driving DLM activity examined in this study. Corresponding parameters displayed in panels in ***B*** are indicated by corresponding panel numbers. Darker backgrounds indicate that significant age-dependent alterations were detected. ***B***, Normalized changes in neurophysiological parameters of the respective motor circuits are plotted as function of percentage of mortality. Changes are presented in terms of the “fold change” from the initial (<1% mortality) value [i.e., log_2_ (value/initial value)], such that +1 indicates doubling, while −1 indicates halving of the value of the parameter. First, the plot at the origin (open symbols, <1% mortality) indicates fold changes compared with young WT 25°C for WT 29°C and *Sod* 23°C young flies. Second, the age trajectories comparing flies of different ages to the youngest flies are indicated in fold changes (filled symbols: WT 25°C, blue; WT 29°C, purple; *Sod* 23°C, red). Panels representing circuit parameters that remain relatively robust across the life span in all three populations are lightly shaded, while parameters that display clear age-dependent alterations in at least one rearing condition are shaded darker.

In this study, we adopted a normalization in time scales of aging progression in accordance with the percentage of mortality (Jones et al., 2014) to facilitate comparisons across the genotypes and conditions that yield drastically different chronological life-span curves (Fig. 1*B*). A graphical overview emerges when comparing the age trajectories of the various motor circuit parameters in different fly populations. Relative to the starting point (i.e., youngest age group), changes (in fold on a semilogarithmic log_2_ scale) in various circuit functions can be compared directly for their relative vulnerability or resilience to aging (Fig. 7).

Our results suggest the operation and integrity of motor circuits fall into the following two general categories: aging resilient (Fig. 7, lighter shading) and aging vulnerable (darker shading). Notably, the elements in the GF pathway that mediate the escape reflex appear more robust and show less functional decline up to the very end of the life span and display milder effects of high-temperature stress or reactive oxygen species (ROS) scavenger disruption, with little impact on SLR and LLR threshold and latency (Fig. 7*B*1,2,4,5). In contrast, substantial changes were seen in the more plastic, use-dependent, functions of these circuit components, including the twin-pulse refractory period of the GF pathway and the habituation of its afferent inputs (Fig. 7*B*3,6,9). Furthermore, motor program-related DLM spike readouts, such as the flight and ECS discharge patterns, showed remarkable age-related modifications (Fig. 7*B*8,9,10,11,12).

#### GF pathway SLRs: GF neuron and downstream elements

The GF pathway with its well described neural elements and transmission properties offers an ideal model system to explore cellular mechanisms of aging and neurodegenerative diseases, as previously reported for its SLR properties (Martinez et al., 2007; Sofola et al., 2010; Zhao et al., 2010; Augustin et al., 2018). Consistently, our characterization of SLRs underlying the escape reflex demonstrates robustly maintained basic mechanisms, for signal propagation from the GF neuron through the PSI and DLM motor neuron to postsynaptic DLMs. Over a large portion of the life span, including the oldest flies (95% mortality), WT flies reared at both 25°C and at 29°C did not show a clear trend of increase or decrease in either SLR latency or threshold, but a decreasing trend in both threshold and latency was noted in *Sod* mutant flies, indicating a consequence of chronic oxidative stress (Figs. 2*C*, 7*B*1,2).

More evident aging-vulnerable alterations were seen in the activity-dependent properties for flies grown under high temperature or ROS stressors. For WT flies reared at 25°C, frequency-dependent characteristics of the SLR, including the twin-pulse RP and FF_30_, were relatively stable with no statistically significant age-dependent changes detected (Fig. 3*A*, Table 1). However, this is not the case for 29°C-reared WT or *Sod* mutant flies since detectable but opposite trends of changes were observed in these two categories of flies in both twin-pulse RP and FF_30_ readouts (Figs. 3*A*, 7*B*3, Table 1), suggesting differential effects on certain vulnerable properties by these two types of stressors (e.g., mechanisms underlying axonal conduction and/or synaptic transmission).

#### GF afferent processing: LLR refractory period and habituation

The processing of afferent signals from upstream circuits activates the GF LLRs (Fig. 7*A*). Similar to SLRs, we found that activity-dependent properties of LLRs (i.e., RP and habituation) were more markedly modified over the life span than the subtle changes in latencies and thresholds (Figs. 2*D*, 3*B*4, 7*B*4,5, Table 1). Given the apparent stability of transmission of the GF neuron and downstream elements, these changes hint at age-dependent plasticity in GF afferents in the brain (Engel and Wu, 1996, 1998), presumably in identified visual (Ache et al., 2019) or mechanosensory (Lehnert et al., 2013; Mu et al., 2014; Blagburn, 2020) inputs.

We observed increasing trends of LLR latency and threshold as flies age (Figs. 2*D*, 7*B*4,5, Table 1). However, although age-dependent alterations observed in LLR latency are statistically significant, they are relatively minor in magnitude, constituting only small fractions in time scale within a 5–20 ms behavioral repertoire for visually or olfactorily triggered GF-mediated escape responses (Engel and Wu, 1992, 1996; Trimarchi and Schneiderman, 1995a; von Reyn et al., 2017).

We also noted that the LLR RP determined by the paired-pulse protocol revealed substantial heterogeneity in the older WT populations examined as indicated by an extremely large spread of data points (50 and 95% mortality at 25°C and 29°C; Fig. 3*B*, Table 1), indicating the stochastic nature of aging progression in GF afferent signal processing.

Importantly, the LLR habituation protocol (Engel and Wu, 1996, 1998) demonstrated the clearest monotonic age-dependent changes in the performance of the GF pathway. In both 25°C-reared and 29°C-reared WT, as well as *Sod*, populations, aged flies habituated much faster compared with younger counterparts (Figs. 4, 7*B*9). Consistent across all populations, these effects were evident for all frequencies tested (1, 2, and 5 Hz; *r*_s_ range, 0.33–0.56; significant *p* values). Furthermore, habituation is commonly considered a simple form of nonassociative learning (Engel and Wu, 2009; Rankin et al., 2009). Our observation that the *Drosophila* GF habituation rate increases monotonically and robustly with age (Figs. 4, 7*B*9) renders this plasticity parameter a potential quantitative index of aging progression for an important aspect of nervous system function.

#### Flight

Throughout most of their life span, WT flies maintained their flight ability (Fig. 1*C*) with stable WBFs (Figs. 1*D*,*F*, 7*B*7), suggesting the presence of effective homeostatic adjustments to overcome aging-associated changes in biomechanical properties of flight muscles and thoracic case. As previously reported (Miller et al., 2008), WBF is stable beyond the median life span despite marked changes in muscle structure and stiffness (see also Chaturvedi et al., 2019). Despite the nearly invariant WBFs, we found the flight pattern generator circuit driving DLM displayed a clear and largely monotonic increase in firing rate with age (Figs. 1*E*, 7*B*8), and a robust phenotypic change in both 25°C-reared and 29°C-reared flies. Conceivably, the age-dependent increases in firing rate could indicate changes in DLMn excitability or flight patterning circuit signal processing among the relevant thoracic interneurons. In either case, this provides a compensatory mechanism to ensure flight ability as aging progresses, namely, increasing the firing rate of muscle Ca^2+^ action potential to retain appropriate intracellular Ca^2+^ levels for powering sustained flight in myogenic stretch-activated DLMs (Pringle, 1978; Gordon and Dickinson, 2006; Lehmann et al., 2013).

#### Electroconvulsive seizure

In tethered flies, high-frequency stimulation across the brain recruits a remarkably stereotypic pattern of nervous system-wide spike discharges accompanied by wing-buzzes (in a fixed succession of ID, paralysis, and DD, cf. Lee and Wu, 2002), reminiscent of the sequence of seizures and paralysis induced by mechanical shock in “bang-sensitive” mutants (Ganetzky and Wu, 1982; Burg and Wu, 2012). Importantly, the spiking associated with the ID and DD is independent of the GF system; during the ECS repertoire, transmission along the escape pathway is blocked (Pavlidis and Tanouye, 1995). Analysis of decapitated flies and Ring-X gynandromorph mosaics has further indicated that both ID and DD pattern generators reside in the thorax, although globally synchronized electric activities and quiescence episodes concurrent with the ID–paralysis–DD repertoire also emerge along the body axis (Lee and Wu, 2002) and across the brain (Iyengar and Wu, 2021). However, the ID and DD are likely derived from separable network origins, as they are independently vulnerable to different ion channel mutations (Lee and Wu, 2006; Kasuya et al., 2019).

In both WT and *Sod* flies, we noted a clear age-dependent decrease in the threshold of ECS-evoked seizure across the life span (Figs. 5, 7*B*10). The monotonic characteristic of this reduction suggests seizure threshold may be considered as another quantitative index of the age progression in nervous system function. Indeed, aged rats reportedly show reduced threshold for kainate-induced seizures (Liang et al., 2007) and elderly humans display increased incidence of seizure disorders (Tallis et al., 1991; Leppik et al., 2006).

ECS discharge spiking patterns exhibit age-dependent trajectories as well (Fig. 7*B*11,12), most dramatically during the ID phase (Fig. 6). Instead of a monotonic trend, ID spiking increases initially with age, peaking at ∼50% mortality, then declines to earlier levels toward the end of the life span (Figs. 6*B*, 7*B*11). It is also intriguing to see that changing ID spiking intensity seemingly provides a forecast for subsequent mortality rate. Indeed, with appropriate lags (11 and 6 d for 25 °C-reared and 29 °C-reared flies), there are high degrees of cross-correlation between the two parameters (Fig. 6*F,G*). It would be interesting to examine this relationship in additional mutants with intrinsically altered neuronal excitability and life span. For example, both hyperexcitable *Shaker* (*Sh*) and hypoexcitable *mle^napts^* (*nap^ts^*) mutants display shortened life spans (Trout and Kaplan, 1970; Reenan and Rogina, 2008) along with drastically altered ECS discharges (Lee and Wu, 2006), flight pattern generation (Iyengar and Wu, 2014; A. Iyengar and C.-F. Wu, unpublished observations), and habituation (Engel and Wu, 1998).

### Distinctive patterns of aging-related changes by high temperature and oxidative stress

The distinct aging trajectories manifested in the functioning of the various motor circuits described above clearly delineate the manners in which high-temperature rearing and oxidative stress, imposed by external environment versus internal milieu, differ in their effects on aging progression in motor functions.

Ambient temperature is a key factor in determining life span in *Drosophila* (Loeb and Northrop, 1917) and other ectotherms. With rearing at high temperature (29°C), life span is shortened by ∼40–50% compared with 25°C, (Fig. 1*B*). Our data support the notion that aging progression is faster at 29°C, but it largely retains the characteristics of the motor performance trajectories of 25°C, as though it proceeds at a “compressed” time scale. Parameters that were age resilient in 25°C-reared flies were robustly maintained in 29°C-reared individuals (SLR and LLR latency and threshold, flight WBF; Fig. 7*B*, lighter shade). Similarly, age-vulnerable parameters generally preserved consistent trends in age-dependent changes when scaled for percentage of mortality. This is true particularly for increased DLM firing frequency during flight, increased LLR habituation rate, and decreased ECS seizure threshold (Fig. 7*B*8–10, darker shade). Remarkably, even the bell-shaped age profile of ID spiking was retained in the 29°C-reared WT population (Fig. 7*B*11). For the remaining parameters, SLR and LLR refractory periods, habituation, and DD spiking, 29°C rearing appeared to intensify their age dependence, leading to considerably steeper trends (Fig. 7*B*3,6,12). Our findings suggest that in the shortened life spans of temperature-stressed individuals, the temporal characteristics of neurophysiological changes is largely retained, albeit “accelerated” according to the degree of life-span compression. Importantly, these observations give justification for the common practice of using 29°C rearing to expedite experiments in *Drosophila* aging studies.

Our findings from *Sod* mutants indicate that oxidative stress exerts strong influences on some physiological parameters with outcomes distinct from the effects of high-temperature rearing. Oxidative stress, resulting from inefficient clearance of metabolic ROS (e.g., superoxide anion and other free radical species), is thought of as a major contributor to aging processes (Harman, 1956, 1981; Finkel and Holbrook, 2000). A key class of ROS scavenging enzymes is the superoxide dismutases, which convert superoxide anions into hydrogen peroxide (Hart et al., 1999; Miller, 2012). In *Drosophila*, the following three Sod enzymes have been identified: intracellular Cu^2+^/Zn^2+^ Sod1 [encoded by *Sod* (Phillips et al., 1989)], mitochondrial Mn^2+^ Sod2 [encoded by *Sod2* (Duttaroy et al., 2003), also known as *bewildered* (Celotto et al., 2012)], or extracellular Sod3 [encoded by *Sod3* (Jung et al., 2011)]. Stress resistance and longevity of the fly are greatly compromised when any of the three is disrupted. Indeed, the *Sod* allele described in this study has no detectable enzymatic activity (Phillips et al., 1989) and exhibits locomotor defects as well as sensitivity to mechanical, paraquat, and heat stress along with greatly shortened life span (Ruan and Wu, 2008).

Across the *Sod* populations, the general trend still holds true in that the identified age-resilient parameters could sustain functionality throughout the life span (GF SLR and LLR latency and threshold, flight WBF; Fig. 7*B*, lighter shade). For several aging-vulnerable properties, *Sod* flies also follow the general profile of age progression albeit with modified amplitude of expression. These are apparent in DLM firing during flight and ID spiking (Fig. 7*B*8,11), and, more notably, in both an accelerated rate of habituation and reduced ECS seizure threshold, closely resembling the corresponding age trajectories in WT counterparts (Fig. 7*B*9,10). Nevertheless, more striking alterations in *Sod* are seen in the activity-dependent properties of the GF pathway, as reflected in the trajectories of the refractory period for both SLR and LLR that are clearly distinct from high-temperature effects (Fig. 7*B*3,6).

A unique feature of the aging trajectories of *Sod* motor functions is the apparent anomaly in some age groups that exhibit unexpected trends in phenotypic extremity. Among different age groups of *Sod* mutants, the youngest age group (<5% mortality) displayed the most conspicuous deviations from the WT counterpart in several categories of motor performance (Fig. 7*B*), including diminished flight ability (Fig. 1*C*), longer SLR latency (Fig. 2*C*), lengthened SLR and LLR RPs (Fig. 3), increased ECS threshold (Fig. 5*B*), and decreased spiking during seizure discharges (Figs. 5*C*, 6*B*). These parameters were often accompanied by a greater spread in measurements among the youngest *Sod* flies (Figs. 3*A*,*B*, 5*B*). Intriguingly, the aged *Sod* flies (70 and 95% mortality) paradoxically performed at levels comparable to their aged WT counterparts (Fig. 7*B*), as though they could outperform the young mutant flies (Iyengar and Wu, 2021). It is possible that secondary mutations in the *red* genetic background contribute to the observed differences between the *Sod* mutants and their WT counterparts, as genetic background effects can have profound effects on specific aging phenotypes (Partridge and Gems, 2007). However, observations from an independently generated loss-of-function *Sod* allele (*Sod^21^*) revealed a similar set of GF transmission phenotypes in young *Sod^21^* flies (<5% mortality), including a retarded SLR latency and a poor ability of SLRs to follow high-frequency stimulation (Ueda et al., 2021). Thus, unidentified modifiers in the background are an unlikely explanation for the respective *Sod* phenotypes reported here, which can be seen even in the youngest adult *Sod* populations.

A likely explanation for this seemingly counterintuitive trend could be the highly stochastic nature of oxidative insults in both the development and function of neural circuits such that a portion *Sod* flies carry more severe but variable in-born defects, compounding the manifestation of aging progression, distinct from aging processes. Therefore, the *Sod* mutant population undergoes a progressive selection over time in favor of healthier individuals, while those with poor circuit performance die off earlier. Consistent with this view, *Sod* mutants reportedly have difficulty eclosing from their pupal case (Phillips et al., 1989; Şahin et al., 2017; Woods, 2017) and the *Sod* life-span curve lacks the earlier plateau phase (Fig. 1*B*) resulting from much higher mortality in young individuals compared with WT populations. This possibility is further highlighted in a recent study demonstrating certain differences in the functional consequences of oxidative stress imposed chronically throughout development in mutant flies and those resulting from acute induction by drug feeding to healthy flies. In WT flies, elevated ROS levels induced by paraquat feeding lead to a distinct collection GF transmission phenotypes compared with *Sod* mutants (Ueda et al., 2021). For example, the SLR latency and refractory period defects in *Sod* mutants are not observed in paraquat-fed WT flies.

### Physiologic hallmarks of aging progression in motor circuits: quantitative biomarkers

It is desirable to search for potential physiological aging indices to completion of existing cellular and molecular biomarkers of aging in *Drosophila*, such as erroneous expression of the transcription factor genes *wingless* and *engrailed* (Rogina et al., 1997), accumulation of glycation end products (Oudes et al., 1998; Baynes, 2001; Jacobson et al., 2010) and heat shock protein (Landis et al., 2004; Tower, 2011), as well as modified functional states of the insulin and target of rapamycin signaling pathways components (Kapahi et al., 2004; Partridge et al., 2011).

Among the various age-dependent trajectories in motor circuit performance, two protocols, LLR habituation and ECS induction, yielded parameters that displayed remarkably consistent aging progressions in normal, high temperature-reared and ROS-stressed populations. Specifically, both acceleration of the habituation rate (Fig. 4) and reduction in ECS seizure threshold (Fig. 5*B*) displayed a relatively uniform, monotonic age trajectory (Fig. 7*B*9,10), providing an unambiguous readout of the circuit performance index along the percentage of mortality axis. It will be particularly interesting to examine whether the age trajectories of these parameters are similarly scaled against life span in long-lived flies such as WT flies under calorie restriction conditions or in *chico* and other insulin pathway mutants. Furthermore, future studies can use these physiological properties to study the relationships between life span and health span in the context of various environmental conditions, pharmacological interventions, or genetic manipulations, which affect longevity.

Future studies will further evaluate their suitability as readily accessible, quantitative indices for more incisive aging assessment in WT and mutant flies. This will be a desirable addition in aging research using a wealth of available mutants, such as insulin signaling pathway mutants that are known to affect life span, under various environmental conditions, pharmacological manipulations, or influences of interacting genes.

## Synthesis

Reviewing Editor: David Schoppik, New York University - Langone Medical Center Decisions are customarily a result of the Reviewing Editor and the peer reviewers coming together and discussing their recommendations until a consensus is reached. When revisions are invited, a fact-based synthesis statement explaining their decision and outlining what is needed to prepare a revision will be listed below. The following reviewer(s) agreed to reveal their identity: NONE.

Hi,

This is a fine paper and I feel we can move forward based on the eLife reviews rather than sending this out again for additional evaluation if you can make the following changes:

1. Reviewer 3 has a point: if the sod mutant wasn’t backcrossed on to the CS background, then when evaluating the comparison between sod to CS one must be conservative with respect to the conclusions. Please do the following: 1) clarify what the background of the sod mutants was in the Methods and 2) if it hadn’t been backcrossed properly (say, 4 generations) on to CS, then please add a paragraph to the Discussion highlighting this fact and referencing the literature that Reviewer 3 raised to make clear what you think the limitations / reservations of genetic background may or may not be.

2. Please cite/discuss your paraquat findings & propose the chico experiments in the Discussion (p24)

## Author Response

1. Significance statement is “Too specialized; please broaden for non-specialists interested in aging generally.”

Following this suggestion, we have re-written the statement.

2. “Clarify what the background of the Sod mutants was in the Methods.”

In the Methods section (P. 4, 3rd para.) we now point out the Sod1 allele is on a red chromosome background (see Phillips et al., 1989, PNAS).

3. “If [the Sod line] hadn’t been backcrossed properly (say, 4 generations) on to CS, then please add a paragraph to the Discussion highlighting this fact and referencing the literature that Reviewer 3 raised to make clear what you think the limitations / reservations of genetic background may or may not be.”

As suggested by reviewer 3, we raise the possibility of background effects on the Sod1 phenotypes, and cite the Partridge & Gems review as suggested in P. 25, 1st para. (the Augustin 2018 paper is cited in a discussion of specific GF properties). Backcrossing the Sod1 allele into the CS background would be beyond our capabilities at this time. However, as we now point out in the discussion (P. 25, 1st para), we have recorded from young Sod21 null mutants (an independent CRISPR-induced mtuation on a white background) which correspond with the < 5 % mortality in this paper (Ueda et al., 2021 microPublication). The key GF short-latency defects: increased latency and decreased high-frequency following are found in both Sod1 and Sod21 mutants.

4. “Please cite/discuss your paraquat findings & propose the chico experiments in the Discussion (p24).”

We have highlighted our discussion of the paraquat feeding experiments in P25, 2nd para. In P26, 2nd para, we now mention that it will be interesting to observe how age-trajectories of various neurophysiological properties scale in long-lived flies, such as chico mutants.
